# Identification of the Different Gene Expression Characteristics from Liver Cirrhosis to Hepatocellular Carcinoma Using Single-Cell Sequencing Analyses

**DOI:** 10.1155/2021/6619302

**Published:** 2021-01-18

**Authors:** Xin Xing, Jian Song

**Affiliations:** ^1^Shanghai Fengxian District Central Hospital, No. 6600, Nanfeng Road, 201499 Shanghai, China; ^2^Department of Radiation Oncology, Renji Hospital, School of Medicine, Shanghai Jiao Tong University, 200127 Shanghai, China; ^3^Institute of Physiological Chemistry and Pathobiochemistry, University of Münster, 48149 Münster, Germany

## Abstract

The occurrence of hepatocellular carcinoma (HCC) is closely related to the chronic inflammation which caused liver fibrosis and cirrhosis, and the interaction between HCC and its microenvironment further drives tumorigenesis. However, the single-cell resolution *in vivo* study is lacking, which limits our molecular understanding of tumour biology in the liver. Here, using published single-cell sequencing technology (scRNA-seq) database, we analyzed the liver microenvironment at high resolution in an unbiased manner and demonstrated the transcriptomic comparison between various cell populations and subpopulations in HCC and cirrhosis tissues. We found that eight genes that are specifically expressed in the endothelial cell and stellate cell of the HCC patients and correlated them with their survival rate, which may provide novel diagnosis and treatment targets for the clinical application.

## 1. Introduction

Hepatocellular carcinoma (HCC) is the most common primary liver cancer, which seriously harms human health. In recent years, the emerging targeted cancer therapy methods have greatly changed the prospects of HCC treatment, but their curative effect varies greatly among different HCC patients [1, 2]. A detailed understanding of the microenvironment of adjacent tissues is of great reference value for better understanding of HCC and the development of new targeted therapies. A unique feature of HCC is that its occurrence and development are closely related to liver fibrosis. More than 80% of HCC develops from fibrosis or cirrhosis of the liver, which is different from most other tumours and organs [3]. Fibrosis of other organs is usually not strongly associated with cancer development, but reactive connective tissue is formed after tumourigenesis. The unique connection between HCC and fibrosis indicates that liver fibrosis in the precancerous environment (PME) of the liver plays an important role in the development of hepatocellular carcinoma [4].

It has been known that the close interaction between tumour cells and liver microenvironment drives tumourigenesis [5]. Although a large number of studies in the liver cirrhosis to HCC have provided *in vitro* evidence or animal model for the role of cancer-associated fibroblasts (CAFs) in promoting HCC, we still lack *in vivo* research evidence [6]. In order to study the role of liver microenvironment in the initiation of HCC, it is necessary to study the interaction of HCC with different components of PME in a physiological manner. Although recent studies in a variety of liver injury models have emphasized the close interaction between tumour cells and the liver microenvironment affected by multiple risk factors for liver damage [7], the physiological interaction of HCC with different components of PME is still elusive. Recently, the emerging single-cell sequencing technology (scRNA-seq) provides a high sensitivity tool and allows us to study low-abundance cell types in health and disease *in vivo* [8, 9]. Also, the high resolution of scRNA-seq is changing our molecular understanding of liver function and the tumour pathogenesis [10]. In oncology research, molecular characterization of tumour cell populations at the single-cell level helps to clarify the evolutionary trajectory of cancer and the time sequence of cancer drivers, which might provide insights into the interaction between tumour cells and paracancerous cells for better cancer intervention [11].

Here, we use scRNA-seq to analyze the liver microenvironment at high resolution in an unbiased manner. Our data reveals the difference between various cell populations and subpopulations in HCC and fibrosis and compares the specific gene expression of different cell subtypes. More importantly, we find genes that are specifically expressed in the endothelial cell and stellate cell of the HCC patients and correlated them with their survival rate, which provides many new diagnosis and treatment targets for the clinical application.

## 2. Methods

### 2.1. Single-Cell mRNA Sequencing Data

NCBI-GEO (https://www.ncbi.nlm.nih.gov/geo/) is a free database of gene profiles and next-generation sequencing, from which single-cell RNA sequencing data of GSE136103 (health and fibrosis) and GSE125449 (HCC) were obtained [11–13]. The two sets of data were all based on preparation on the 10x Genomics platform, 4 and 3 cases of noncancerous tissues from healthy and cirrhotic patients, respectively, and 16 cases of HBV-related HCC tissues.

### 2.2. Single-Cell mRNA Sequencing Data Analysis

R package Seurat was used for data analyses. In the quality control, we removed the UMI counts less than 500 and the doublets. Also, cells were filtered with the percentage of mitochondrial genes (>10%). Samples from healthy, cirrhotic, and HCC tissues were normalized with sctransform method and integrated with the reciprocal principal component analysis (PCA) (https://satijalab.org/seurat/v3.1/integration.html) [14]. The integrated dataset was further subjected to PCA and considered for cluster analyses with a unified manifold approximation and projection (UMAP, version 0.2.6.0) in a resolution of 0.2.

Nonmalignant cells were extracted, and the first 10 clusters were selected with UMAP analysis. We annotated the cells based on known cell lineage-specific marker genes such as T cells (CD2 and TRAC), B cells (CD79A and MS4A1), plasma cells (IGHG1), endothelial cells (PLVAP, VWF, ACKR1, and CDH5), stromal cells (COL1A2, PDPN, DCN, and LUM), macrophages (CD14, CD163, MS4A7, and CSF1R), and epithelial cells (KRT19, KRT18, and F2).

### 2.3. Single-Cell mRNA Sequencing Analysis Tool

Seurat (version 2.3.4) was used to fuse single-cell sequencing data from different sources, and treatments, data normalization, and clustering were performed to find differentially expressed genes [14]. Pseudotime analysis was performed using Monocle (version 2.10.1), showing the trajectory of epithelial cells and stromal cells in the fibrosis and HCC liver [15]. To detect the differential expression genes (DEGs), volcano plots in R package ggplot2 (version 3.3.2) were used.

### 2.4. The Survival Rate and Gene Expression Analysis

The survival rates were analyzed by a log rank analysis of 364 HCC patients using TCGA database as described previously [16]. And the gene expression in HCC and normal liver was analyzed using TCGA and GTEx database, respectively. The survival and gene expression analyses were both performed by using an online tool-GEPIA2 (http://gepia2.cancer-pku.cn/#index).

## 3. Results

**Figure 1:**
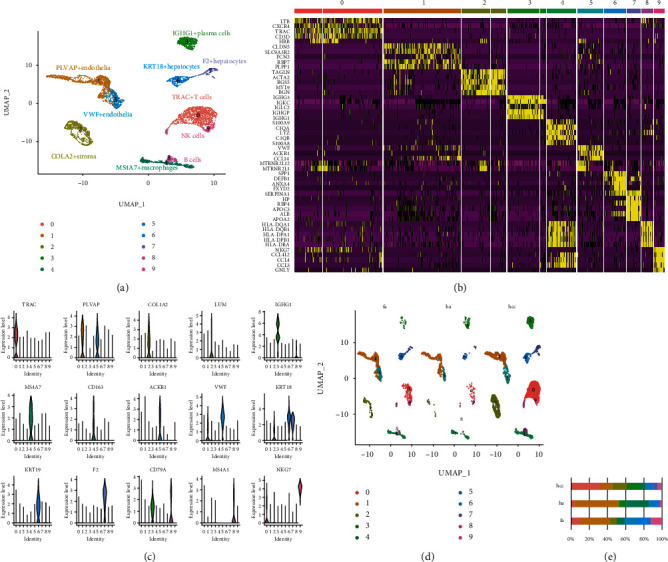
Single-cell RNA-seq analysis in the liver sample from healthy, fibrosis, and HCC patient. (a) UMAP diagram describes the 10 main cell types in the integrated liver biopsy samples. (b) Heat map annotated the different genes of the 10 main cell types. (c) Violin plots show the expression and distribution of marker genes. (d) Single-cell data of healthy (ha), fibrosis (fa), and HCC (hcc) patients. (e) Bar graphs quantify and compare the proportion of single-cell data of healthy (ha), fibrosis (fa), and HCC (hcc) patients.

Unsupervised dimensionality reduction and hierarchical clustering analysis were performed by UMAP, and 10 main cell types were established, namely, TRAC+ T cells (c0), PLVAP+ or VWF+ endothelial cells (c1, 5), COLA2+ stromal cells (c2), IGHG1+ plasma cells (c3), MS4A7+ macrophages (c4), KRT18+ or F2+ hepatic cells (c6, 7), MS4A1+ B lymphocytes (c8), and NKG7+ NK cells (c9) (Figure 1(a)). Each cell type has been successfully annotated by known marker genes (Figures 1(b) and 1(c)), and these clusters are distinctly identified in the samples of healthy, fibrosis, and HCC patient (Figure 1(d)). As the source data of healthy, fibrosis, and HCC samples were all derived from 10X Genomics analysis, we performed integration processing using sctransform. Compared to standard log-normalization, sctransform effectively removes technically driven variation while preserving biological heterogeneity, which can easily switch between RNA, protein, cell hashing, batch-corrected/integrated, or imputed data. Comparing the samples of healthy, fibrosis, and HCC patient (Figure 1(d)), we could see the similarities of clustering after the sctransform integration. The clustering distance of UMAP cells is based on the similarity of gene expression profiles. The clustering images of liver fibrosis and hepatocellular carcinoma are close, indicating that the gene expression profiles of the cells contained in the two have a certain degree of similarity. We compared the proportion of the classified liver cells and found that healthy and fibrotic-derived cells contain more B cells, T lymphocytes, macrophages, and plasma cells but contain fewer endothelial cells and NK cells. This trend becomes more pronounced in the hepatocellular carcinoma samples (Figure 1(e)).

**Figure 2:**
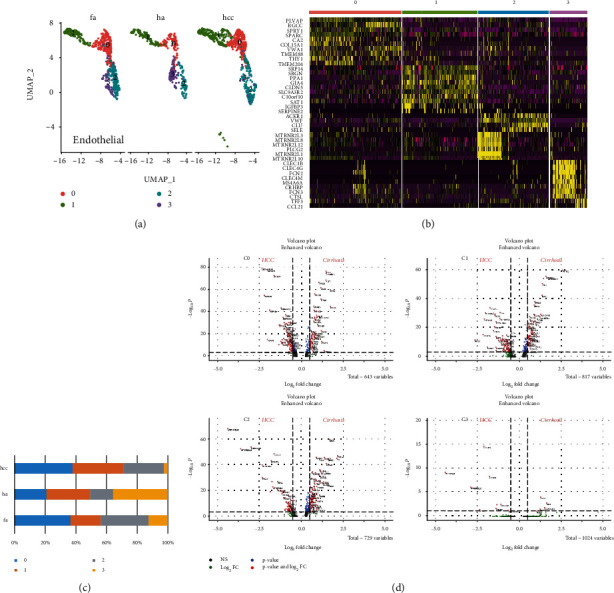
Subcluster analysis and quantification of the liver endothelial cells. (a) UMAP diagram depicts 4 subtypes of the endothelial cells. (b) Heat map annotated differential genes of 4 endothelial cell subtypes. (c) Bar graphs quantify and compare the proportion of endothelial cell subtypes of healthy, fibrosis, and HCC patients. (d) Volcano plots show the DEGs between fibrosis and HCC in 4 subtypes.

To further examine the molecular characteristics at the individual cell level, we performed a subpopulation analysis of endothelial cells. The UMAP map with a resolution of 0.25 showed at least 4 different subpopulations (Figure 2(a)), and the top10 marker gene was found (Figure 2(b)). At the same time, we compared the proportion of endothelial cell subpopulations in the HCC with that of the healthy and liver fibrosis patients (Figure 2(a)). CCL21+ endothelial cells (c3) were significantly reduced in hepatocellular carcinoma. In contrast, both PLVAP+ and VWF+ endothelial cells in the fibrosis and HCC samples were increased compared with the healthy cells, indicating that inflammation as well as angiogenesis happened in cirrhosis and malignant liver (Figure 2(c)). Analysis of differential gene expression in each subgroup revealed the unique transcriptional profiles of the respective endothelial cell subtypes that are derived from liver fibrosis and hepatocellular carcinoma (Figure 2(d)).

**Figure 3:**
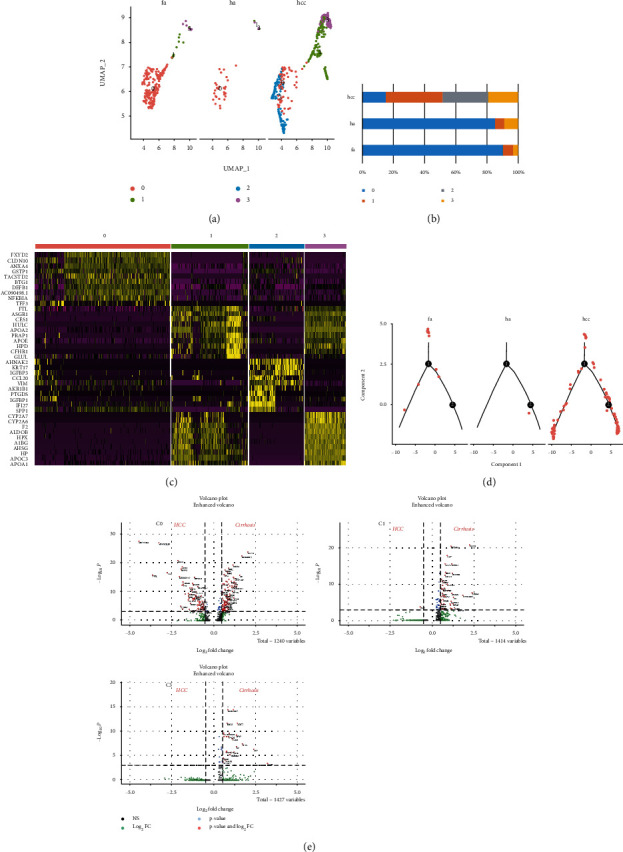
Subcluster analysis of the epithelial cells. (a) UMAP diagram depicts 4 subtypes of the epithelial cells. (b) Bar graphs quantify and compare the proportion of epithelial cell subtypes of healthy (ha), fibrosis (fa), and HCC (hcc) patients. (c) Differential genes of 4 epithelial cell subtypes. (d) Analysis of the trajectory of cluster 1 in the healthy (ha), fibrosis (fa), and HCC (hcc) state. (e) Volcano plots show the DEGs between fibrosis and HCC in 3 epithelial cell subtypes; C2 exists only in the HCC.

In order to study the relationship between simulated liver fibrosis and HCC origin, we first performed fusion and subgroup analysis on all hepatocytes derived from healthy people, patients with liver fibrosis and HCC (Figures 3(a)–3(c)). We found that the c1 subgroup has a significant increase in HCC. Then, using the feature of single-cell sequencing to allow single-cell trajectory analysis, using Monocle to perform pseudotime analysis on the c1 subpopulation, we firstly merged the results of single-cell sequencing including healthy people, patients with liver fibrosis and HCC. According to the characteristics of the gene expression of a single cell, it is arranged in a simulated time sequence to show the development trajectory of the cell. The results of the trajectory analysis suggest that HCC has great heterogeneity (Figure 3(d)). Analysis of differential gene expression for each subgroup (except c2) revealed the unique transcriptional configuration potential of epithelial cells derived from liver fibrosis and hepatocellular carcinoma (Figure 3(e)).

**Figure 4:**
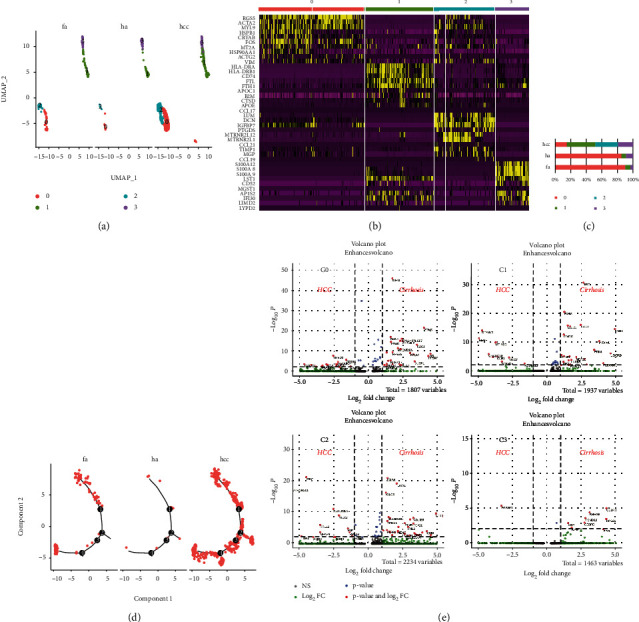
Subcluster analysis of the macrophages and the stroma cells. (a) UMAP diagram depicts the subclustering of macrophages and stroma cells. (b) Heat map annotated the different genes of the subcluster cell types. (c) Bar graphs quantify and compare the proportion of subclusters of healthy, fibrosis, and HCC patients. (d) Analysis of the trajectory of cluster 0 in the healthy, fibrosis, and HCC state. (e) Volcano plots show the DEGs between fibrosis and HCC in 4 subtypes.

Also, we firstly performed fusion and subgroup analysis on the macrophages and stromal cells derived from healthy people, patients with liver fibrosis, and HCC patients (Figures 4(a) and 4(b)). Comparing the proportion of subgroups, we found a consistent increase of c0 subgroup from the heathy to the fibrosis and HCC group (Figure 4(c)). Using the stellate cell marker—RGS5, we classified the stromal cluster 0 as hepatic stellate cells (HSCs) and noticed that compared with liver cirrhosis, the proportion of HSC in HCC tissue was further increased (Figure 4(c)). Trajectory analysis shows many bifurcations in HSCs development, which is pronouncedly increased in the HCC group (Figure 4(d)). Analysis of differential gene expression for each subgroup revealed the unique transcriptional configuration potential of stromal cells derived from liver fibrosis and hepatocellular carcinoma (Figure 4(e)).

**Figure 5:**
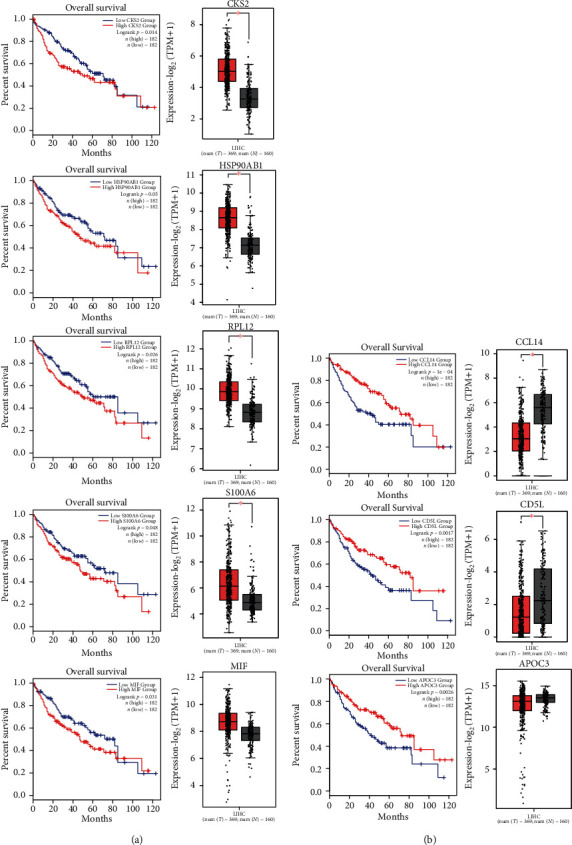
Impact of the DEGs between fibrosis and HCC in the HCC patients. (a, b) Kaplan-Meier analysis of overall survival according to the gene expression in HCC patients from the TCGA dataset. The *p* value was calculated by the log-rank test. (a) The high expression of CKS2, HSP90AB1, RPL12, S100A6, and MIF predicts poor prognosis. Analysis of mRNA expression in HCC compared to normal liver tissue. ^∗^*p* < 0.05. (b) The low expression of CCL14, CD5L, and APOC3 predicts poor prognosis. Analysis of mRNA expression in HCC compared to normal liver tissue. ^∗^*p* < 0.05.

Based on TCGA and GTEx database, we performed the survival rate and gene expression analysis based on the selected integrating gene expression (Figure 5). We found 5 genes including CKS2, HSP90AB1, RPL12, S100A6, and MIF that were associated with significantly shorter overall survival (OS) in HCC patients with high expression compared to those with low expression (Figure 5(a)). In addition, the mRNA expression level of CKS2, HSP90AB1, RPL12, and S100A6 was significantly higher in HCC tissues compared to normal tissues, indicating those 4 genes might be considered the oncogenes. In contrast, we found other 3 genes including CCL14, CD5L, and APOC3 that were associated with significantly shorter OS in HCC patients with low expression compared to those with high expression (Figure 5(b)). And the level of CCL14 and CD5L mRNA expressions was significantly decreased in HCC tissues, revealing CCL14 and CD5L as the potential tumour-suppressor genes. The biological functions of those identified genes need further investigation in order to explore the new biomarkers for HCC diagnosis and the new potential targets for HCC therapy.

## 4. Discussion

The liver microenvironment related to liver cancer has hardly been studied with single-cell resolution, which limits our molecular understanding of liver function and disease biology. The recent emergence of sensitive single-cell RNA sequencing methods allows us to study cell types and their disease pathogenesis in health and tumour-related diseases. Using scRNA-seq, we analyzed here the transcriptome of liver cells from healthy, fibrosis, and HCC patients, at high resolution and in an unbiased manner. Our analyses (1) show that the cell types and their proportions in the liver develop in order, from the healthy to the fibrotic and the malignant liver; (2) reveal the subdivision of endothelial, epithelial, and stromal cells and demonstrate a high heterogeneity that are caused by tumourigenesis; and (3) determine the DEGs in the cell clusters that are sequentially developed from the healthy to the fibrotic and then to the malignant samples and indicate some of the DEGs as the potential novel prognosis markers of survival rate.

On the whole, the process from healthy tissues to liver fibrosis and then to HCC is a process of increasing immune cells, especially T cells, which is related to the inflammatory damage in liver cirrhosis and the immune response caused by tumour tissue. On the other hand, NK cells are significantly reduced in the HCC. It is known that liver NK cells have special phenotypes and functions that are different from peripheral blood and spleen NK cells which can suppress the tumour progression through the TRAIL pathway [17]. The loss of NK cells accelerates the tumour progression in the HCC. Also, we observed a significant increase in B cells and plasma cells in HCC tissues, and a large number of tumour-related macrophages have also been found. This result is consistent with the published single-cell sequencing of HCC immune cells [18]. Although we also did a subtype analysis in this study, since other studies have already had detailed analyses, we did not continue to study here. The current discussion mainly focuses on the subtype analysis and gene expression of nonimmune cells.

From liver cirrhosis to HCC, the main two types of endothelial cells are continuously reduced, and this reduction is mainly caused by tissue cirrhosis and the damage to the vasculature. However, from the perspective of endothelial cell subtypes, there is a visible formation of tumour-related blood vessels. The acceleration of tumour growth creates a hypoxic microenvironment, which means that the formation of new blood vessels in the tumour is a process mediated by hypoxia stimulation and growth factors [19]. From a transcriptomic perspective, tumour endothelial cells upregulate angiogenesis-related genes, such as CD99, KLF10, and ADAMTS4, while losing scar tissue-related Notch molecule HES4 and antigen presentation molecule HLA-DRA. Vascular cells not only bring nutrition to the tumours; the interaction between endothelial hepatocytes and HSCs may contribute to the formation of HCC tumour microenvironment (TME) [20].

Compared to the heathy controls, the stromal cell population is dramatically increased in the cirrhosis and HCC group. Focusing on the HSCs, which is the stromal cluster 0 and RGS5+ [12], we noticed that HSC proportion was further enhanced in the HCC tissue compared to the cirrhosis. However, the trajectory study indicates a large heterogeneity in the HSCs of the HCC tissue, suggesting a high complexity of HSCs under HCC condition. On the other hand, the failure of characterizing the DEGs from those HSCs of HCC shows the chaos of the tumour HSCs, which definitely requires further studies.

In summary, we analyzed in this study the differences between various cell populations and subpopulations in HCC and fibrosis, in a high-resolution and unbiased manner, and compared the specific gene expression of different cell subtypes. More importantly, we found that eight genes are specifically expressed in the endothelial cell and stellate cell of the HCC patients and correlated them with their survival rate, which might provide many new diagnostic and therapeutic targets for clinical applications.

## Data Availability

The datasets and code generated or analysed in this study are available from the corresponding author upon reasonable request.
